# Chromatogram-level fusion of FID and MS signals in GC × GC for quantitative volatilomics: workflow design and impact on pattern recognition

**DOI:** 10.1007/s00216-026-06324-5

**Published:** 2026-02-20

**Authors:** Andrea Caratti, Angelica Fina, Fulvia Trapani, Simone Squara, Erica Liberto, Qingping Tao, Daniel Geschwender, Chase Heble, Stephen E. Reichenbach, Carlo Bicchi, Chiara Cordero

**Affiliations:** 1 Dipartimento di Scienza e Tecnologia del Farmaco, Università Di Torino, Via Giuria 9, 10125 Turin, Italy; 2 GC Image LLC, Lincoln, NE USA; 3 Computer Science and Engineering Department, Lincoln, NE USA

**Keywords:** Comprehensive two-dimensional gas chromatography, Parallel detection, Data fusion, Quantitative volatilomics, UT fingerprinting, Image pattern recognition

## Abstract

Volatilomics is an emerging discipline aimed at characterizing volatile metabolites in diverse matrices. Recently, comprehensive two-dimensional gas chromatography (GC × GC) coupled with parallel flame ionization detection (FID) and mass spectrometry (MS) has gained attention for combining accurate quantification with unambiguous compound identification. Traditionally, FID and MS data are processed independently. This study addresses their integration by merging FID and MS chromatograms into a single fused chromatogram, enhancing pattern recognition during template matching and enabling large-scale quantitative volatilomics. Feature matching is guided by MS spectral similarity, minimizing mismatches and extracting FID responses for robust quantification. The contribution discusses the workflow designed to obtain combined detector signals and the challenges posed by dual parallel detection operated under both thermal and differential-flow modulation, dual-parallel second-dimension column configurations, and differences in acquisition frequency between detectors. From an application standpoint, chromatogram fusion directly responds to emerging analytical needs in volatilomics—supporting quantitative, high-throughput analysis across extended time frames through the FID channel, while ensuring the mandatory MS confirmation required for the reliable identification of fragrance allergens and regulated compounds. The resulting fused chromatogram consolidates complementary detector information within a single multidimensional chromatogram, improving data consistency, interpretability, and throughput. Overall, chromatogram-level fusion represents a key step toward integrated, multimodal analytical platforms for robust and scalable volatilomics workflows.

**Graphical abstract:**

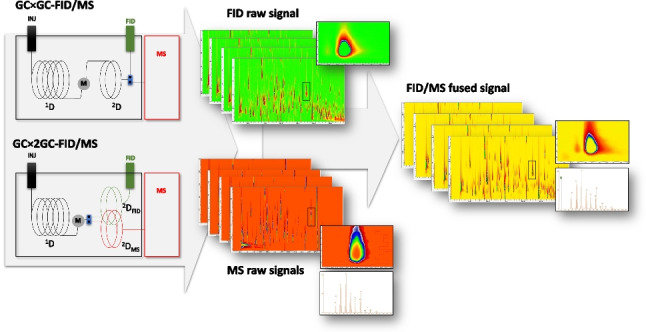

**Supplementary Information:**

The online version contains supplementary material available at 10.1007/s00216-026-06324-5.

## Introduction

Volatilomics, the comprehensive investigation of volatile metabolites in complex matrices, has rapidly emerged as a critical analytical field across diverse domains such as food and fragrance science, environmental monitoring, and clinical metabolomics [1, 2]. The volatile metabolome encodes essential information about sample composition, authenticity, origin, quality, and safety attributes. To fully exploit this information, advanced analytical platforms are required that not only deliver high separation power but also provide reliable identification and robust quantification. In this context, comprehensive two-dimensional gas chromatography (GC × GC) has become the technique of choice, offering enhanced separation capacity and multidimensional selectivity [3, 4].

When comprehensive GC × GC is hyphenated with parallel detection, the analytical platform can take advantage of complementary detectors operating simultaneously. Each detector encodes different, and often non-overlapping, dimensions of chemical information, from universal responses (e.g., flame ionization detection, FID or thermal conductivity detector, TCD) to molecular fingerprints (e.g., mass spectrometry, MS) or element-selective signals (e.g., sulfur chemiluminescence detector, SCD or nitrogen-phosphorus detector, NPD) [5, 6]. By combining these perspectives, parallel detection increases the overall information content of a single run. Among the possible configurations, the combination of FID and MS has become one of the most widely adopted strategies, as it unites two core requirements in volatilomics: accurate quantitative profiling and reliable qualitative identification [7].

Regarding accurate quantitative profiling, FID is widely recognized as the detector of choice, as it is universal, responds proportionally to the mass of carbon-containing compounds combusted in the flame, and offers outstanding sensitivity, wide linearity, and low susceptibility to matrix effects. These features make it particularly suitable for quantitative analysis in flavour and fragrance applications, where analytes span broad concentration ranges [8]. In addition, FID offers the advantage of employing response factors (RF) and relative response factors (RRF) for accurate quantification. In practical applications, the RF compensates for the fact that equal molar amounts of different substances often do not generate identical peak areas, and RRFs are used to normalize analyte signals against an internal standard, thereby improving quantification accuracy across a broad range of compounds [9–11]. In recent years, an important innovation has been introduced by De Saint Laumer and colleagues, who developed a method to predict RRFs in GC-FID directly from molecular formulae and combustion enthalpies [10]. This approach opens the way to multitarget quantification, allowing large sets of compounds to be quantified from a single calibration curve without requiring individual calibration standards. Such predictive quantification strategies are especially attractive in volatilomics, where the number of relevant compounds often exceeds the feasibility of compound-by-compound calibration (i.e., external calibration). However, the universality of FID is also its weakness: the lack of selectivity means that interfering compounds cannot be distinguished from target analytes, leading to potential overestimation or ambiguous quantification. In complex natural matrices such as fragrances or food volatilomes, where co-elution is frequent due to the high chemical dimensionality [12], this limitation is critical [13, 14]. A recent study demonstrated the feasibility of reconstructing TIC response factor surfaces for non-targeted quantification in GC × GC–MS. By systematically modeling and interpolating TIC response factors, predictive surfaces were generated to estimate quantitative responses for compounds outside the calibration set. This strategy enables multitarget quantification without individual calibration standards, thereby extending the quantitative capacity of GC × GC–MS for large-scale, non-targeted analyses and overcoming one of the main limitations of MS-based quantification in complex volatilomes [15]. This is particularly relevant considering that, beyond quantification, MS detection provides a complementary strength through its reliable qualitative identification. By recording molecular fragmentation patterns, MS produces a real molecular fingerprint that allows compound identification through comparison with libraries and retention index databases [16]. In contexts such as allergen detection in fragrances or authenticity verification of foodstuffs, MS is mandatory [17]. Moreover, in cases of co-elution, MS can resolve overlapping peaks by monitoring characteristic ions for each analyte, thereby discriminating compounds that would otherwise be indistinguishable. However, MS quantification is hampered by several factors: a narrower linear dynamic range compared to FID, signal drift due to source contamination, and inter-instrument variability. In GC × GC–MS, this limited dynamic range is further affected by band compression—spatial in thermal modulation and temporal in differential flow modulation—which amplifies the difficulty of maintaining linearity across the wide signal-to-noise ratio (S/N) encountered over multiple modulations and runs. Even with internal standardization, reproducibility across large-scale studies remains challenging. Thus, while MS is essential for qualitative identification, FID remains superior for robust quantification [18].

From an application standpoint, these advantages are particularly relevant in the food and fragrance studies. Here, product quality, authenticity, and safety are tightly linked to quali-quantitative changes in the volatile and semi-volatile fraction. For example, subtle differences in cultivar, geographic origin, or processing can translate into perceptible aroma differences that define consumer preference and market value. At the same time, regulatory compliance often requires the monitoring of specific allergenic or restricted compounds, which can only be unambiguously identified and/or confirmed for the identity by MS or MS/MS signatures. Despite the clear advantages of using FID and MS in parallel, the practical implementation of this strategy in GC × GC is not straightforward. The two detectors inherently generate independent data streams, each requiring its own preprocessing, peak detection, and elaboration, which makes the workflow redundant and time-consuming. As a result, the full potential of combining accurate FID-based quantification with unambiguous MS-based identification has long been hindered by the complexity of dual data processing. This study is part of a long-term project aiming at obtaining reliable qualitative and quantitative routine analyses using a single GC × GC run with simultaneous FID and MS detection, and collecting their signals in a unique set of compatible and significant data and processing. For these reasons, fusion or combination into a single data stream of FID and MS chromatograms addresses this challenge by generating a single chromatogram that is directly subjected to processing. Within this fused signal, the FID response (single channel detector), reliably informing about analyte amount in the sample, and the qualitative/quantitative information from MS (multi-channel detector) are both preserved, so that the information from the two streams can be easily extracted. This approach eliminates redundant workflows while enhancing the robustness and reliability of quantitative volatilomics.

A conceptually related precedent can be found in the work of Cordero et al., where the fusion of variable ionization energy MS data streams (i.e., Tandem Ionization™ [19]), combining hard and soft ionization modes, was shown to extend the dynamic range of detection and quantification and improve classification performance in cocoa volatilome studies. Although that study addressed fusion within a single detector type, it clearly demonstrated how integrating complementary signals can enhance sensitivity and robustness. Building on this principle, the present work aims to advance the concept further: rather than merging signals from the same detector under different acquisition modes, the focus here is on integrating data from fundamentally different detectors. The fusion of FID and MS chromatograms in GC × GC is expected to provide an even more comprehensive chemical representation. In this perspective, a further step could be represented by the combination with Tandem Ionization™ data fusion, yielding greater sensitivity and a more detailed characterization of the sample.

In the present work, we build upon these premises by systematically evaluating the opportunities and challenges associated with FID/MS chromatogram fusion in GC × GC. Specifically, we investigate how this approach performs across different analytical configurations, including GC × GC with flow modulation and FID/QMS detection, GC × 2GC with thermal modulation and FID/QMS detection, and GC × GC with thermal modulation coupled to FID/TOF–MS operated with Tandem Ionization™. By comparing these scenarios, we aim to assess both the strengths and the critical limitations of chromatogram-level fusion, providing insights into its applicability for quantitative volatilomics in diverse experimental contexts.

## Materials and methods

### Chemicals and solvents

Pure standards of n-alkanes (from *n*-C9 to *n*-C25) for Linear Retention Indices (*I*^T^_S_) calibration were from Merck Life Sciences Srl (Milan, Italy). Pure standards (or isomers mixtures) of volatile allergens listed in Supplementary Table 1 – ST1, hazelnuts’ potent odorants for identity confirmation, pure standards of 1,4-dibromobenzene, 4,4-dibromobiphenyl, and α/β-thujone used as Internal Standards (ISTDs), and solvents (cyclohexane, dichloromethane, and diethyl phthalate) were all from Sigma-Aldrich (Milan, Italy).

### Volatile allergens reference solutions and raw hazelnut samples

Standard Stock Solutions (SS) of volatile allergens were prepared at a concentration of 10 mg/mL in dichloromethane or cyclohexane and stored at − 18 °C. The Model Mixture (MMix) stock solution was prepared by mixing suitable amounts of SS at a final concentration of 200 mg/L in cyclohexane. Calibration levels covered were as follows: 1–5–10–20–50–100 mg/L, ISTDs were at a final concentration of 50 mg/L. Standard reference solutions for purity evaluation (by 1D-GC-FID) were prepared from SS at a nominal concentration of 100 mg/L in cyclohexane. More details on allergen mixture can be found in Stilo et al*.* [20].

Raw hazelnuts from crop 2022 were provided by Soremartec Italia Srl (Alba, Cuneo, Italy). Samples were stored at − 18 °C away from UV exposure until analysis. The variables characterizing the sample set included four different cultivars/geographical origins (Tonda Gentile Trilobata –TGT, Tonda Gentile Romana –TGR, Akçakoca mix –AKC, Giresun mix –GIR) informing about the phenotype expression in the volatilome. Hazelnuts were then stored until 12 months after their harvest, and samples were collected and analyzed at 0, 6, and 12 months. Storage, impacting sensory quality and off-flavor development, was at 5 °C and 65% of equilibrium relative humidity under vacuum. Insights on the samples adopted for this application are available in Squara et al*.* [21].

### GC × GC platforms: configurations and analytical conditions

The various parallel detection scenarios employed to implement the concept of data fusion are schematically summarized in Table 1, which reports the corresponding column configurations, split ratio between the two detectors, MS and FID operative conditions, temperature programs, and modulation parameters.

**Table 1:** Column configurations and instrumental parameters for the three analytical set-ups (A, B, and C), including column characteristics, split ratio, detector configuration, oven temperature programs, and modulation parameters

	**Colum configuration**	**Split ratio and capillaries**	**Detector information**	**Oven programming**	**Modulation parameters**
**Set-up A FM-GC × GC-QMS/FID**					
* Hazelnut*	^1^D DB-HeavyWax™ 20 m × 0.18 mm *d*_c_ × 0.18 µm *d*_f_ He @ 0.4 mL/min—constant flow ^2^D DB17 1.8 m × 0.18 mm *d*_c_ × 0.18 µm *d*_f_ He @ 10 mL/min—constant flow Injector in pulsed-split mode at 250 kPa until 2.5 min with a 1:5 split ratio	FID/MS split ratio 70:30 to MS: 0.35 m, 0.10 mm *d*_*c*_ to FID: 1.1 m, 0.18 mm *d*_*c*_	Fast quadrupole, mass range of m/z 40–250, sampling frequency 28 Hz FID 300 °C base temperature, H_2_ flow 40 mL/min, the air flow was 350 mL/min, N_2_ flow 40 mL/min, sampling frequency 200 Hz	40 °C (2′) to 130 °C (0′) @ 4 °C/min, and to 260 °C (10′) @ 8 °C/min	*P*_*M*_ = 2.5s pulse time: 250 ms
* Allergens*	^1^D DB-1 20 m × 0.18 mm *d*_c_ × 0.18 µm *d*_f_ He @ 0.5 mL/min—constant flow ^2^D OV17 1.8 m × 0.18 mm *d*_c_ × 0.18 µm *d*_f_ He @ 8 mL/min—constant flow Injector in split mode with a 1:20 split ratio	FID/MS split ratio 70:30 to MS: 0.4 m, 0.10 mm *d*_*c*_ to FID: 1.1 m, 0.18 mm *d*_*c*_	Fast quadrupole, mass range of m/z 40–250, sampling frequency 28 Hz FID 300 °C base temperature, H_2_ flow 40 mL/min, the air flow was 350 mL/min, N_2_ flow 40 mL/min, sampling frequency 200 Hz	60 °C (0.72′) to 280 °C (7.16′) @ 5.58°C/min	*P*_*M*_ = 3 s pulse time: 150 ms
**Set-up B TM-GC × GC-TOFMS/FID (Tandem Ionization)**					
	^1^D SolGel-Wax 30 m × 0.25 mm* d*_*c*_ × 0.25 µm *d*_*f*_ He @ 2 mL/min—constant flow ^2^DOV1701 1.8 m × 0.18 mm* d*_*c*_ × 0.18 µm *d*_*f*_ He @ 2 mL/min—constant flow Injector in split mode with a 1:20 split ratio	FID/MS split ratio 70:30 to MS: 0.7 m, 0.10 mm *d*_*c*_ to FID: 1.1 m, 0.18 mm *d*_*c*_	Bench TOF-Select™ system featuring Tandem EI (70 eV, 12 eV), mass range of m/z 35‐350, sampling frequency 50 Hz per channel FID 300 °C base temperature, H_2_ flow 40 mL/min, the air flow was 350 mL/min, N_2_ flow 40 mL/min, sampling frequency 200 Hz	60 °C (1′) to 280 °C (10′) @ 4°/min	*P*_*M*_ = 5 s pulse time: 300 ms
**Set-up C TM-GC × 2GC-QMS/FID**					
	^1^D DB-5MS 30 m × 0.25 mm* d*_*c*_ × 0.25 µm *d*_*f*_ He @ 2.3 mL/min—constant flow ^2^D DB1701 1.4 m × 0.1 mm* d*_*c*_ × 0.1 µm *d*_*f*_ He initial head pressure: 302 kPa—constant flow Injector in split mode with a 1:10 split ratio	FID/MS split ratio 1:1to MS: DB1701 1.4 m × 0.1 mm* d*_*c*_ × 0.1 µm *d*_*f*_ + 0.20 m × 0.1 mm *d*_c_ to FID: DB1701 1.4 m × 0.1 mm* d*_*c*_ × 0.1 µm *d*_*f*_	Fast quadrupole, mass range of m/z 40–240 (5 to 40 min) and m/z 40–330 (40 min till the end of the analysis), sampling frequency 28 Hz FID 280 °C base temperature, H_2_ flow 40 mL/min, the air flow was 350 mL/min, N_2_ flow 40 mL/min, sampling frequency 100 Hz	60 °C (1′) to 240 °C (0′) @ 3 °C/min to 280 °C (5′) @ 25 °C/min	*P*_*M*_ = 4 s pulse time: 300 ms

### Set-up A: GC × GC with flow modulator, parallel detection FID/QMS

GC × GC analyses with reverse-inject differential flow modulation were run on an Agilent 7890 A GC unit coupled to an Agilent 5977B HES (High Efficiency Source) fast quadrupole MS detector (Agilent Technologies, Little Falls, DE, USA) operating in EI mode at 70 eV and a fast FID detector. The MS was tuned using the HES Tune option. The full‐scan acquisition was made at the *fast scanning* rate of 12,500 amu/s to obtain a spectrum generation frequency of 28 Hz, with a mass range of m/z 40–250. The MS source was set at 230 °C, the quadrupole at 150 °C, and the transfer line temperature was at 280 °C. Parallel detection was achieved with a FID set at 300 °C base temperature, the H_2_ flow was 40 mL/min, the air flow was 350 mL/min, the make-up (N_2_) at 40 mL/min, and the sampling frequency at 200 Hz. Modulation was by differential-flow modulator via the reverse-inject dynamics; it was realized on the Capillary Flow Technology™ CFT (G4573A, Agilent Technologies). After the ^2^D column, the flow was split using a three-way unpurged capillary microfluidic splitter (G3181B, Agilent Technologies). The connections toward the MS and FID consisted of deactivated silica capillaries (Agilent Technologies) a calibrated to obtain split ratio of 70:30 FID/MS. The bleeding capillary consisted of deactivated silica; dimensions were calculated using a validated calculator to balance flows into the plate and avoid any loss see details in Table 1[22].

#### Analytical condition for allergens application

Injections of the calibration mixtures, as well as those for Retention Indices (*I*^T^) determination, were carried out with a 4513 A auto injector sampler (Agilent, Little Falls, DE, USA) under the following conditions: injection mode: split, split ratio: 1:20 for calibration mixtures and 1:50 for *n-*alkanes, injection volume 2 µL, injection temperature 280 °C. The modulation period (*P*_M_) was 3 s and the pulse time was 150 ms. The column configuration was the following: ^1^D DB-1 (100% Dimethylpolysiloxane, 20 m × 0.18 mm *d*_c_ × 0.18 µm *d*_f_) coupled with a ^2^D OV17 ((50%-phenyl)-methylpolysiloxane; 1.8 m × 0.18 mm *d*_c_ × 0.18 µm *d*_f_) both from Agilent Technologies—J&W (Little Falls, DE, USA). The carrier gas was helium at a nominal flow of 0.5 mL/min along the ^1^D column and 8 mL/min along the ^2^D column. The oven temperature program was the following: 60 °C (0.72 min) to 280 °C (7.16 min) @ 5.58 °C/min.

#### Analytical condition for hazelnuts application

The divinylbenzene/carboxen/polydimethylsiloxane 2 cm SPME fiber was from Supelco (Bellefonte, PA, USA) and used for HS-SPME sampling. The fiber was installed on a multipurpose sampler, model MPS-2 (Gerstel, Mülheim a/d Ruhr, Germany). The standard in-fiber procedure [23] was adopted to preload the Internal Standard (IS) onto the fiber before sampling. A 5.0 µL solution of IS (α/β -thujone at 100 mg L^−1^ in diethyl phthalate) was placed into a 20 mL glass vial and subjected to HS-SPME at 50 °C for 5 min. After the IS loading step, the SPME device was exposed to 100 mg of hazelnut in headspace glass vials (20 mL) for 50 min at 50 °C. Extracted analytes were recovered by thermal desorption of the fiber into the S/SL injection port of the GC system for 5 min. The injector operated in pulsed-split mode at 250 kPa until 2.5 min with a 1:5 split ratio. The *P*_M_ was 2.5 s and pulse time was 250 ms. The column configuration was the following: ^1^D HeavyWax™ (100% polyethylene glycol—PEG; 20 m × 0.18 mm *d*_c_ × 0.18 μm *d*_f_) coupled with a ^2^D DB17 ((50%-phenyl)-methylpolysiloxane; 1.8 m × 0.18 mm *d*_c_ × 0.18 μm *d*_f_) both from Agilent Technologies. The carrier gas was helium at a nominal flow of 0.4 mL/min along the ^1^D column and 10 mL/min along the ^2^D column. The oven temperature program was the following: 40 °C (2 min) to 130 °C (0 min) @ 4 °C/min, and to 260 °C (10 min) @ 8 °C/min.

### Set-up B: GC × GC with thermal modulator, detection FID/TOF MS with Tandem Ionization™

GC × GC analyses with thermal modulation were performed on an Agilent 7890B GC unit coupled with a Bench TOF-Select™ system (Markes International, Llantrisant, UK) featuring Tandem Ionization™. For the purposes of this study, *hard* ionization at 70 eV was set for identity confirmation while lower electron ionization energy was set at 12 eV. The ion source and transfer line were set at 280 °C. The MS optimization option was set to operate in Tandem Ionization™ with a mass range between 35 and 350 m/z; data acquisition frequency was 50 Hz per channel. Parallel detection was achieved with a FID set at 300 °C base temperature, the H_2_ flow was 40 mL/min, the air flow was 350 mL/min, the make-up (N_2_) was 40 mL/min, and the sampling frequency was 200 Hz. The system was equipped with a two-stage KT 2004 loop thermal modulator (Zoex Corporation, Houston, TX) cooled with liquid nitrogen controlled by Optimode^TM^V.2 (SRA Instruments, Cernusco sul Naviglio, MI, Italy). The hot jet pulse time was set at 300 ms, *P*_M_ was 5 s, and cold-jet total flow was progressively reduced with a linear function from 40% of Mass Flow Controller (MFC) at initial conditions to 5% at the end of the run. The column set was configured as follows: ^1^D DB-1 (100% Dimethylpolysiloxane; 30 m × 0.25 mm *d*_c_ × 0.25 µm *d*_f_) coupled with a ^2^D OV1701 (86% polydimethylsiloxane, 7% phenyl, 7% cyanopropyl; 2 m × 0.1 mm *d*_c_, 0.10 µm *d*_f_), both from J&W (Agilent Technologies, Little Falls, DE, USA). After the ^2^D column, the flow was split using a three-way unpurged capillary microfluidic splitter (G3181B, Agilent Technologies). The connections toward the MS and FID consisted of deactivated silica capillaries (Agilent Technologies) 0.7 m × 0.1 mm *d*_c_ and 1.1 m × 0.18 mm *d*_c_ respectively, resulting in a split ratio of 70:30 FID/TOF MS.

Injections of the calibration mixtures, as well as those for *I*^T^ determination, were carried out under the following conditions: split/splitless injector in split mode at 250 °C, split ratio 1:20 for calibration mixtures and 1:50 for *n-*alkanes, and injection volume 2 μL. The carrier gas was helium at a constant flow of 2 mL/min. The oven temperature program was from 60 °C (1 min) to 280 °C (10 min) @ 4 °C/min.

### Set-up C: GC × 2GC with thermal modulator, dual-secondary column/dual detection FID/QMS

GC × GC analyses with thermal modulation were carried out on an Agilent 6890 GC unit coupled to an Agilent 5975 MS detector operating in EI mode at 70 eV (Agilent Technologies, Little Falls, DE, USA). The full‐scan acquisition was made at the *fast scanning* rate of 12,500 amu/s to obtain a spectrum generation frequency of 28 Hz, with a mass range of m/z 40–240 (5 to 40 min) and m/z 40–330 (40 min till the end of the analysis). The MS source was set at 230 °C, the quadrupole at 150 °C, and the transfer line temperature was at 280 °C.

Parallel detection was achieved with a FID set at 280 °C base temperature; the H_2_ flow was 40 mL/min, the air flow was 350 mL/min, the make-up (N_2_) was 40 mL/min, and the sampling frequency was 100 Hz. The system was equipped with a two‐stage KT 2004 loop‐type thermal modulator (Zoex Corporation, Houston, TX, USA) cooled with liquid nitrogen and controlled by Optimode™ V.2 (SRA Instruments, Cernusco sul Navigo, Italy). The hot‐jet pulse time was set at 300 ms and the *P*_M_ at 4 s, and the cold‐jet total flow was progressively reduced with a linear function from 35% of the mass flow controller at initial conditions to 5% at the end of the run. The hot‐jet temperature was programmed from 220 °C to 290 °C at 3 °C/min.

The column set was configured as follows: ^1^D DB5–MS (30 m × 0.25mm *d*_c_ × 0.25 µm *d*_f_) from J&W (Agilent Technologies, Little Falls, DE, USA) and an inert three‐way “T‐Inert” splitter (Agilent G3184‐60065) to split the effluent of the first column into the two parallel ^2^D‐columns two identical DB1701 columns (1.4 m × 0.1 mm *d*_c_ × 0.10 µm *d*_f_) from J&W (Agilent Technologies, Little Falls, DE, USA). The first 0.6 m of the two columns was wrapped together in the loop‐type thermal modulator slit. One of them was directly connected to the FID, and the outlet of the second one was connected to a deactivated capillary (0.20 m × 0.1 mm *d*_c_) with a SilTite™ µ‐union (SGE, Ringwood, Australia), and then to the MS source. Injections of the allergens calibration mixtures, as well as those for *I*^T^ determination, were carried out by using an autosampler ALS 7683B (Agilent), in split mode, with a split ratio of 1:10, at 280 °C. The carrier gas was helium at a constant flow (initial head pressure: 302 kPa). The oven temperature program was from 60 °C (1 min) to 240 °C (0 min) at 3 °C/min, and then to 280 °C (5 min) at 25 °C/min.

### Data processing

The data were acquired by Enhanced MassHunter (Agilent Technologies, Little Falls, DE, USA) for the Set-up A, TOF-DS software (Markes International,Llantrisant, UK) for the Set-up B, and Agilent MSD ChemStation G1701EA E.02.02.1431 (Agilent Technologies) for the Set-up C. 2D data were processed by GC Image® GC × GC Edition Software, Release2.9 (GC Image, LLC Lincoln NE, USA).

## Result and discussion

Data fusion provides a powerful strategy to overcome the intrinsic limitations of using FID and MS separately. By integrating the universal and highly reproducible response of FID with the qualitative specificity of MS, the fused signal enables simultaneous access to accurate quantitative information and reliable compound identification within a single dataset. This dual assurance simplifies workflows by eliminating redundant data processing, while at the same time enhancing robustness, sensitivity, and confidence in peak assignment. Moreover, the generation of a single fused chromatogram supports more consistent peak detection and image-based pattern recognition, facilitating large-scale comparative studies. Beyond technical simplification, fusion offers a more comprehensive chemical representation, as complementary detector information is preserved and can be extracted when needed. Taken together, these advantages underline the transformative potential of chromatogram fusion in volatilomics, especially in contexts where reliability, scalability, and regulatory compliance are critical. The subsequent paragraphs describe the workflow adopted for chromatogram fusion. The results are critically discussed with respect to misalignments and the potential for improved quantification and inter-batch data transferability.

### Data fusion procedural steps

For this study, the GC Image™ software (GC Image LLC) was used to implement the workflow. The fusion procedure consisted of well-defined steps, designed to ensure channel alignment, standardized data representation, and final integration of FID and MS signals into a single dataset.

Step 1—FID and MS chromatogram/image generation and alignment

FID and MS chromatograms/images were first imported into the software for preprocessing and channel alignment. The appropriate phase shift was applied to align the two channels, followed by resampling with identical values for both MS and FID signals, ensuring a coherent number of data points per unit time. At this stage, both channels were prepared for further conversion and integration, but no data fusion was yet performed.

Step 2—Generation and import of the pseudo-MS FID file

The FID channel was exported as a text-formatted MS file, where mass spectra—although absent in the case of FID—were represented as structured text containing virtual *m/z* values (set to 0, as no true ions are present) and the corresponding intensity values. This configuration enables further elaboration of the FID signal as if it were a real MS dataset.

The conversion can be easily implemented within the software with two steps: exporting the FID as a text-formatted MS file and then re-importing it as an MS dataset. No additional processing steps such as phase shift, baseline correction, or resampling were applied during this re-import action. The resulting chromatogram/image represents the FID signal in a format directly comparable to MS data.

Step 3—Chromatograms fusion

The FID pseudo-MS image obtained in Step 2 was then merged with the MS chromatogram/image. In this fused chromatogram/image, the FID intensity signal is encoded within the fragmentation pattern as a virtual fragment at *m/z* 0, while the original MS fragmentation data remain preserved in their respective *m/z* channels. The outcome is a single fused chromatogram/image that combines the robust quantitative response of the FID with the qualitative spectral information of the MS, providing an integrated dataset for subsequent processing and interpretation.

Step 4—2D peak detection and spectral information inspections

Peak detection in the fused chromatogram proceeds as in conventional GC × GC–MS workflows, with the additional advantage that both FID-derived quantitative information and MS spectral fingerprints are available within the same dataset. The presence of the virtual *m/z* 0 fragment, which encodes the FID intensity, does not interfere with spectral inspection or library matching, as it can be excluded during search. Consequently, peak recognition and spectral identification remain fully reliable while benefiting from the complementary contribution of the FID signal.

### FID/MS raw data misalignment: causes, effects, and strategies to approach them

After defining the general steps for chromatogram fusion, the critical issue becomes understanding what should actually be merged. Image-level fusion in the presence of retention time misalignments may generate artificial peaks that do not correspond to real analytes, thereby compromising both quantitative and qualitative interpretation. Differently from what was demonstrated by Cordero et al*.*, where fusion was applied to chromatograms originating from two equivalent data streams (i.e., variable ionization energy MS) [24], the present case involves the integration of detectors with intrinsically different acquisition characteristics. FID and MS operate with distinct sampling frequencies and may produce subtle but systematic discrepancies in peak profiles. Even minimal misalignments can result in artificial peak duplication in the fused chromatogram, undermining both peak recognition and quantitative accuracy. Therefore, the success of this fusion strategy critically depends on achieving precise synchronization of the FID and MS data streams, supported by accurate calibration of the analytical set-up and robust alignment algorithms.

In the literature, retention time misalignment has frequently been observed and reported, particularly in long-term studies where instrumental variability accumulates over extended time frames. For instance, Stilo et al*.* [25] demonstrated that in extra-virgin olive oil fingerprinting, column aging, batch-to-batch variability, and modulation instabilities contributed to distortions in 2D peak patterns, ultimately complicating both targeted and untargeted template matching. Similarly, in parallel dual-column configurations, as described by Nicolotti and co-workers [26], systematic absolute and relative retention time shifts were observed between FID and MS channels. These deviations, often in the order of tenths of a second in the second dimension, are partly due to the distinct flow conditions of each 2D branch and partly to the inherently different acquisition rates of FID (typically > 100 Hz) and MS (20–100 Hz). When represented as 2D images, these unequal sampling frequencies translate into a pixel-level misalignment that can amplify artifacts in fused chromatograms if left uncorrected [27].

Taken together, the literature points to two main scenarios in which retention time misalignment arises. In the first case, observed in long-term or large-scale studies, the observed misalignment can be attributed to the combined influence of column phase degradation, batch-dependent variations, and gradual instrumental drift. As a result, retention time shifts are not constant and may vary across the chromatographic space and over time, complicating reproducibility and template-based approaches. In the second case, which occurs in dual-channel acquisitions, misalignment is essentially determined by the analytical set-up itself. Here, retention time offsets between detectors remain constant within a batch of analyses, but the degree of shift is compound-specific rather than uniform across all analytes, reflecting differences in molecular properties and interactions with the stationary phase.

To exemplify the type of misalignment arising from the analytical set-up itself, we considered the three set-ups described in the experimental section, using a test mixture of fragrance allergens as model compounds. The investigated configurations were as follows: (i) GC × GC with flow modulation and parallel detection, in which FID and QMS signals were acquired after a post-column split; (ii) GC × GC with thermal modulation and parallel detection, coupling FID and TOF MS operated under Tandem Ionization™, also after a post-column split; and (iii) GC × 2GC with thermal modulation and a dual-secondary column set-up, enabling dual parallel detection with FID and QMS. To quantify the extent of detector misalignment under these different conditions, a subset of target analytes was selected to span the chromatographic space and cover a broad range of volatilities and polarities. Relative misalignment values, expressed as a fraction of the ^2^D retention time using the FID as reference, the peak width at the base (*ω*_b_), the peak width at half height (*ω*_h_) for the three set-ups are reported in Table 2.

**Table 2:** Relative misalignment (RM) values, expressed as a fraction of the ^2^D retention time using the FID as reference, together with the average peak widths at the base (*ω*_b_) and at half height (*ω*_h_) for all analytes, expressed in milliseconds (ms). Peak width values represent the mean between FID and MS measurements for each analyte across the three set-up

		**Set-up A**			**Set-up B**			**Set-up C**		
**Compound Name**	**Lit. I**^***T***^	**Relative misalignment**	***ω***_**h**_	***ω***_**b**_	**Relative misalignment**	***ω***_**h**_	***ω***_**b**_	**Relative misalignment**	***ω***_**h**_	***ω***_**b**_
Benzaldehyde	942	54	373	634	16	335	570	174	106	180
Limonene	1023	136	207	351	30	265	450	400	69	117
Citronellol	1211	69	312	530	42	337	572	42	247	420
Cinnamyc Alcohol	1275	24	316	538	13	469	797	41	314	534
Eugenol	1335	26	391	665	36	366	622	17	331	562
β-Damascone (*Z*)	1402	53	269	457	46	351	596	28	153	260
Camphor	1410	53	265	450	35	314	533	58	141	239
Isoeugenol (*E*)	1424	28	358	608	31	364	619	32	330	561
Coumarin	1441	15	439	746	20	500	850	1262	337	573
α-Santalol	1660	46	260	442	53	367	624	27	236	401
Benzyl Benzoate	1737	24	299	508	29	403	684	3	312	530
Benzyl Cinnamate	2130	16	337	573	21	512	869	12	471	800
*Mean*	*-*	*45*	*319*	*542*	*31*	*382*	*649*	*175*	*254*	*431*

As shown in Table 2, the average absolute misalignment is very small in the first two set-ups, with mean values of 45 ms (Set-up A) and 31 ms (Set-up B). These figures are substantially lower than the mean baseline peak widths (542 ms for Set-up A and 649 ms for Set-up B) and therefore fall within intrinsic chromatographic variability. This limited effect can be explained by the experimental arrangement: in both configurations, the detector split is performed after the second-dimension column, and the two detector branches are implemented as the short inert transfer capillaries that connect the ^2^D outlet to FID and MS. Any difference in linear carrier velocity between the branches therefore acts only over these short capillary lengths, producing only minor retention offsets that remain negligible relative to the 2D peak breadth. Consequently, such misalignments can be generally ignored in routine data-fusion processing for these set-ups without compromising feature recognition or quantitative reliability.

**Fig. 1:**
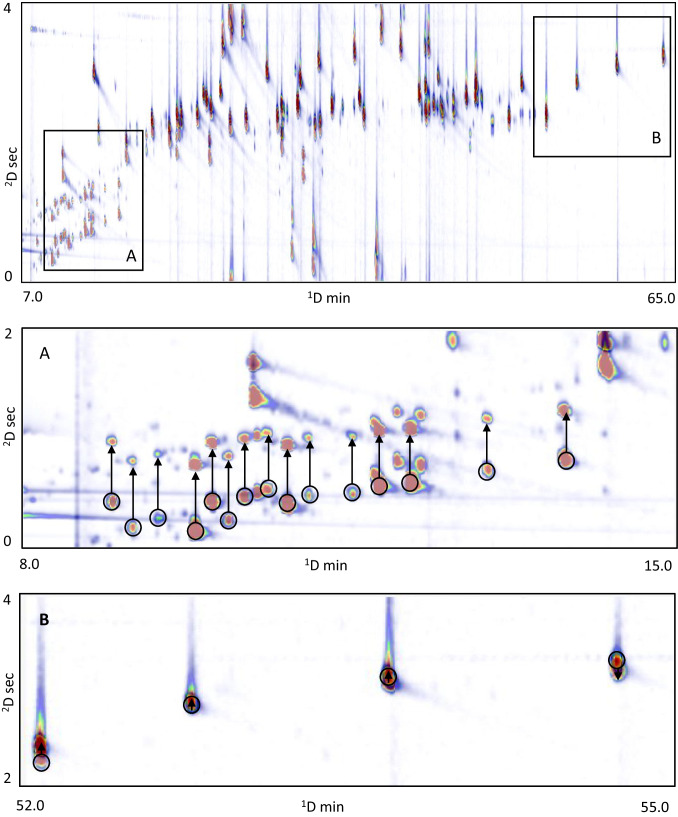
Overlayed visualization of two misaligned chromatographic regions (A and B), displayed with transparency to highlight ^2^D retention time shifts. In A, a more pronounced misalignment is observed (tracked by black arrows), as the compounds involved exhibit low *K* values, while in B, the misalignment appears less pronounced

By contrast, in Set-up C, the average misalignment is considerably larger (175 ms, ranging from 3 ms for benzyl benzoate to 400 ms for limonene). This pronounced effect arises from the dual-secondary configuration itself: the parallel second dimensions operate under different outlet pressure conditions (FID at atmospheric pressure versus MS under vacuum), which induce systematic differences in effective relative carrier velocity across the two ^2^D columns and produce compound-dependent retention offsets. This results in compound-dependent shifts, with analytes early eluted in ^2^D, characterized by lower retention factors (or *capacity factors)* (*K*) being more affected, whereas species with higher *K* values show improved alignment, as already reported by Nicolotti et al*.* [26]. However, the extent of misalignment is not uniform across the entire range of analytes. It tends to be more pronounced for compounds eluting within the preset modulation period and exhibiting low *K* values, then progressively decreases for species with higher *K*, before increasing again for those affected by the wrap-around phenomenon. In the latter case, as exemplified by coumarin, showing a relative misalignment of about 1262 ms, the estimation of the *K* becomes uncertain, since the exact number of modulation cycles the compounds were subjected to after transfer to the second dimension cannot be reliably determined. In this case, the misalignment is large enough to require explicit correction prior to image-level fusion. Figure 1 shows the entities of misalignments between the FID and MS channels observed in Set-up C. Insights on the differential impact of flow-regime and pressure-drop in such configurations are reported in reference literature [26, 28, 29].

In this context, Reichenbach and co-workers [26] have shown that such issues can be systematically addressed by quantifying both absolute and relative misalignment in dual-secondary column and dual-detector GC × GC systems. They highlighted that the extent of misalignment may vary across the chromatographic plane: deviations are not uniform for all analytes but depend on their position in the second dimension and on their retention characteristics. To overcome these distortions, the authors introduced polynomial transformation models capable of mapping retention times between channels, thereby correcting for systematic offsets and restoring accurate alignment [30]. Importantly, their workflow integrates template-based image pattern recognition and registration, which allows the chromatographic image under investigation to be matched and adapted to the reference template through the selected transformation functions. This process ensures that the two detector images become spatially aligned, enabling direct correspondence between FID and MS responses across the chromatographic plane.

**Fig. 2:**
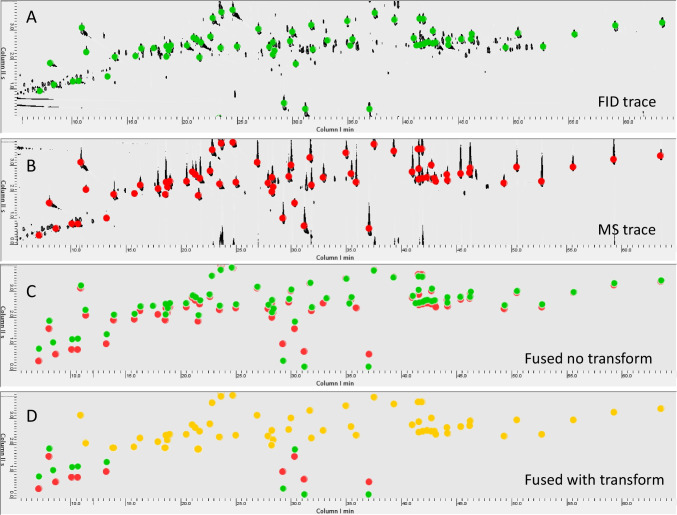
Chromatogram overlay showing the misalignment between FID (**A**) and MS (**B**) detector signals before and after the application of the second-order polynomial transformation. Colored circles indicate the 2D peaks centroids in FID (green) or MS (red) pattern. The fused chromatogram in **C** shows the overlay of the 2D peaks patterns, while in **D,** the global 2^nd^ order polynomial transform effectively compensates for retention time shifts in peaks (yellow circles) eluting between 2 and 4 seconds in the ^2^D, resulting in full alignment of FID and MS peak centroids. Pseudo-colorization is adjusted to highlight targeted peaks patterns

Building on this concept, we applied the same approach to the analyses performed under Set-up C. Figure 2 illustrates the degree of misalignment before and after the application of the second-order polynomial transformation. The image clearly shows that, following the template-based matching step, the transformation effectively compensates for all retention shifts observed in peaks eluting in the second dimension between 2 and 4 s, where the FID and MS peak centroids become aligned. Conversely, for compounds eluting between 0 and 2 s—where the misalignment was more severe—the second-order transformation alone was not sufficient to fully correct the retention offsets.

**Fig. 3:**
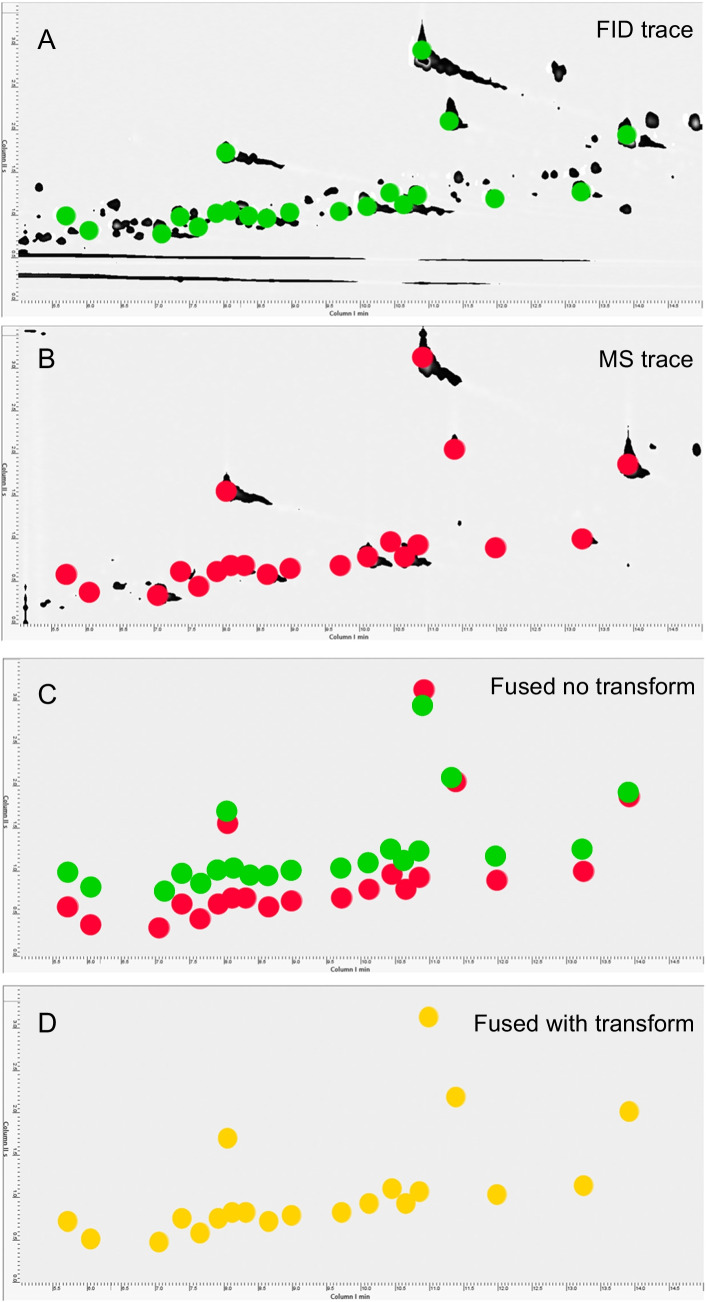
Localized alignment applied to the cropped chromatographic region (0–15 min) following the zonal correction strategy proposed by Squara et al. [31]. The images show 2D peak patterns from the chromatographic area before and after the local transformation. In particular, **A** is the original FID trace (green circles for targeted peaks centroids), **B** is the MS TIC trace (red circles), **C** is the misaligned fused chromatogram with 2D peaks centroids in the original position, and **D** is the transformed and registered chromatogram with re-aligned patterns (yellow circles). The approach successfully compensates even the most severe misalignments and restores full correspondence between FID and MS peaks

To address this, the chromatogram was further adapted using a zonal alignment strategy introduced by Squara et al. [31], who demonstrated that local, region-specific transformations can significantly improve alignment accuracy in cases of complex, non-linear retention distortions. In their approach, the chromatographic space is divided into smaller zones according to the local retention behavior of analytes, and independent polynomial transformations are applied to each region to correct for localized deviations. In accordance with this principle, the chromatogram was cropped to isolate the section between 0 and 15 min, where the most pronounced misalignment occurred, and the local transformation was applied to that region. Figure 3 shows the chromatographic pattern before and after this correction.

This localized alignment strategy proved to be highly effective, successfully compensating even the most severe misalignments observed in the early-eluting region. After the application of the zonal correction, the FID and MS chromatographic features were brought into full correspondence, demonstrating that this approach is capable of restoring accurate peak matching even under challenging analytical conditions. Such combined methodological and computational strategies enable the simultaneous exploitation of complementary detector signals without generating artificial peaks or compromising reliability. These findings reinforce the view that detector fusion, while ambitious, becomes a powerful strategy when supported by robust alignment and registration protocols, as it preserves both the quantification power of FID and the structural selectivity of MS within a single, coherent GC × GC data stream. Beyond alignment correction, other advantages of detector fusion include extending the dynamic range for quantification—by integrating the complementary responses of the two detectors within a single dataset—and increasing confidence in untargeted analyses, particularly when dealing with batch effects or retention shifts over large time frames. These aspects will be further discussed in the following sections.

### MS/FID extended linearity

Linearity represents a fundamental requirement for reliable quantitative analysis, particularly in volatilomics where analyte concentrations often span several orders of magnitude. MS typically provides stable linearity and high sensitivity at low concentration levels, making it indispensable in situations where coelutions occur and unambiguous identification of target compounds is required. For examples, in the fragrances field, this selectivity ensures that even trace-level allergens can be accurately detected and quantified despite the presence of interfering compounds. However, the dynamic range of MS remains relatively limited, often covering no more than two to three orders of magnitude, which constrains its applicability for analytes present at higher concentrations. By contrast, FID is characterized by an exceptionally broad dynamic range, extending over several orders of magnitude with a nearly linear response and deviations within 10% even for major constituents of natural extracts. This robustness enables accurate quantification of compounds present at high or very high concentrations, which are frequently encountered in raw fragrances, natural extracts, and essential oils used in cosmetic and perfume formulations.

Belhassen et al. clearly demonstrated this complementarity by showing that QMS provided reliable quantification in the low concentration range (2–100 mg/kg), while FID maintained excellent linearity at higher concentrations (100–10,000 mg/kg). The combined use of the two detectors thus enabled accurate quantification across four orders of magnitude within a single analysis, significantly reducing the need for multiple dilutions [32]. Similarly, Stiloet al*.* confirmed that both thermal modulation and differential flow modulation GC × GC platforms can deliver comparable quantitative performance, with linear responses obtained across wide concentration ranges and correlation coefficients consistently exceeding 0.995. Importantly, their study highlighted that the intrinsic differences between modulation strategies did not compromise detector performance, as FID maintained a stable linear response regardless of modulation type. These findings underline the robustness of FID as a quantitative detector and further support its role in extending the effective dynamic range when used in parallel with MS [20].

Within the framework of chromatogram fusion, these complementary features are directly inherited in a single data stream: the fused chromatogram preserves the wide dynamic range of FID for robust quantification while embedding the selectivity and qualitative discrimination of MS, particularly essential in cases of coelution. This dual advantage not only improves quantification reliability across diverse concentration ranges but also enhances the robustness and scalability of volatilomics workflows, particularly in regulatory contexts where both sensitivity and breadth of coverage are required.

To systematically evaluate calibration quality and further substantiate these considerations, calibration curves were established for a representative set of analytes covering a broad range of volatilities and polarities, and analyzed across all three instrumental set-ups. Two-dimensional normalized volumes (independent variable, *y*), referring either to Target Ion responses or to the FID signal for fully resolved peaks, were plotted against normalized concentrations (dependent variable,* x*), and the coefficient of determination (*R*^*2*^) was adopted as the quality parameter. To note, for all calibration points and replicates, it was verified that the heteroscedastic distribution of residuals never exceeded the ± 20% of error [29]. Table 3 lists the *R*^2^obtained for the different analytes across the three instrumental set-ups, for both FID and MS detectors. In italic *R*² values below the 0.995 limit are considered acceptable according to the reference methodology [29]. The corresponding calibration curves, along with their associated *R*^2^ values, are provided in Supplementary Table 2—ST2.

**Table 3:** Coefficients of determination (*R*^2^) for selected analytes across the three instrumental set-up, obtained using both FID and MS detectors. In Italic are reported those values below the acceptability limit (i.e., 0.995) [32]

	**Set-up A**		**Set-up B**			**Set-up C**	
**Compound**	**FID**	**MS**	**FID**	**70eV**	**12eV**	**FID**	**MS**
Benzaldehyde	0.998	*0.994*	1.000	0.988	1.000	0.999	0.995
Limonene	0.996	0.99	1.000	0.999	0.999	1.000	0.996
Citronellol	0.998	*0.993*	1.000	0.998	0.999	0.999	0.996
Cinnamyc Alcohol	0.997	0.998	0.999	0.998	1.000	0.995	0.997
Eugenol	0.998	0.998	1.000	0.998	0.997	0.998	0.996
β-Damascone (*Z*)	0.998	*0.991*	0.999	0.998	0.999	0.999	0.995
Camphor	0.998	0.997	1.000	0.996	0.995	0.999	*0.993*
Isoeugenol (*E*)	0.998	0.998	1.000	1.000	0.998	0.996	0.996
Coumarin	0.997	0.995	1.000	0.996	0.998	1.000	0.997
α-Santalol	0.996	*0.994*	0.998	0.999	1.000	0.997	0.999
Benzyl benzoate	0.997	0.997	0.999	0.999	1.000	0.999	0.995
Benzyl cinnamate	0.997	0.998	1.000	0.998	*0.994*	0.995	0.996

This dataset provides a direct comparison of the quantitative performance of the two detectors across a chemically diverse panel of analytes. Except for a few isolated cases where slightly lower coefficients of determination were obtained, the FID exhibited comparable or even superior linearity to MS for most analytes. This confirms its robustness for quantitative purposes, especially when the concentration range extends over several orders of magnitude.

When considering the FID/Tandem Ionization™ configuration (Set-up B), the complementary nature of MS signals acquired at 12 eV and 70 eV further enhances quantitative reliability. The combination of “soft” and “hard” ionization allows the exploitation of a wider dynamic range: the 70 eV channel provides higher sensitivity (LOQ < 0.1 mg/L) but shows limited linearity for some high-abundance compounds, while the 12 eV acquisition maintains a more stable response across a broader range of concentrations.

As for the FID, although the calibration curves in this study were limited to a maximum concentration of 100 mg/L, the observed behavior in all three set-ups is consistent with its well-known capability to preserve linearity over a considerably wide range of concentrations.

**Fig. 4:**
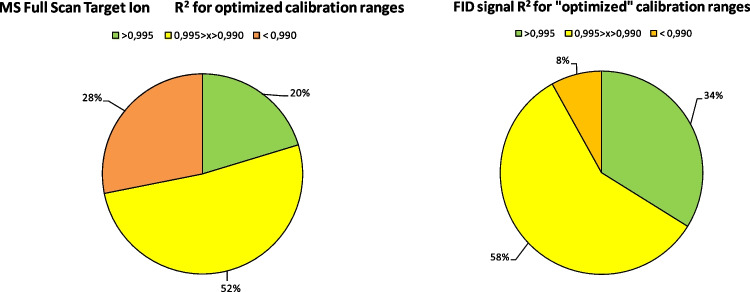
Summary of coefficient of determination distribution within all target analytes; colors indicate the ranking based on acceptability criteria fixed a priori (see text for details)

To further illustrate these performance differences, set-up C was selected for a detailed evaluation of detector linearity across 54 fragrance allergens (for isomer mixtures, just major compounds were included in the testing). The *R*^2^ was computed for each analyte, and the overall calibration performance was visualized in two pie charts summarizing the *R*^2^ distribution. Figure 4 shows these distributions, categorized by acceptability: “good” (*R*^2^ > 0.995), “acceptable” (0.990 < *R*^2^ ≤ 0.995), and “critical” (*R*^2^ < 0.990), with color coding for clarity—green for good, yellow for acceptable, and orange for critical.

The comparison between MS and FID reveals a notable shift in calibration performance. The proportion of critical *R*^2^ values (*R*^2^ < 0.990) decreases from 28% in MS to 8% in FID, indicating improved linearity with FID. *R*^2^ values in the acceptable range (0.990–0.995) remain relatively stable, increasing slightly from 52 to 58%. Meanwhile, the fraction of good *R*^2^ values (*R*^2^ > 0.995) rises by 14%, from 20 to 34%, further highlighting FID’s capability to maintain a broader linear dynamic range.

From a broader perspective, the integration of MS and FID information through data fusion ultimately translates into a more efficient quantitative workflow. The extended dynamic range afforded by the complementary detector responses enables accurate quantification across wide concentration intervals, minimizing the need for multiple dilutions and injections. Moreover, when combined with the use of predicted RRFs, this strategy allows the implementation of multi-target quantification through a single calibration curve, even in the absence of pure reference standards [33]. This approach not only streamlines the analytical process—reducing sample preparation time and data complexity—but also maintains quantitative accuracy and reproducibility across chemically different analytes.

### Chromatographic fingerprinting and FID/MS data fusion

Chromatographic fingerprinting represents a powerful strategy for feature recognition and sample comparison in GC × GC. Its effectiveness, however, strongly depends on the stability and comparability of chromatographic patterns across multiple analyses. This aspect becomes particularly critical in studies performed over extended time frames or across large sample sets, where retention time shifts and other sources of misalignment are more likely to occur.

As already discussed in the previous sections, in multidetector GC × GC workflows, the simultaneous acquisition of signals from complementary detectors such as FID and MS offers a unique opportunity to exploit the strengths of each system. While MS data alone generally provide robust and information-rich inputs for pattern recognition, FID signals—despite their superior quantitative linearity and sensitivity—lack the spectral dimension necessary to reliable track features. Consequently, when the two detectors are processed independently, the FID channel becomes the limiting factor for reliable feature tracking and automated template-based analysis. Moreover, treating them separately requires maintaining two distinct processing workflows, complicating data management and interpretation.

By contrast, data fusion offers a clear advantage for downstream processing: it allows feature matching to benefit from the spectral constraints of MS while permitting quantitative data extraction directly from the same fused chromatogram. The simultaneous availability of both detectors’ information significantly enhances template matching, enabling the reliable tracking of both targeted and untargeted peaks. This capability is particularly valuable in large-scale volatilomics studies, where dedicated software is used to automatically apply *feature templates* across multiple chromatograms involving hundreds of analytes that must be tracked across batches, crops, or processing conditions [34].

When MS information is incorporated, spectral fingerprints introduce an additional constraint that guides the matching process [35]. As demonstrated by Reichenbach et al., the use of Computer Language for Identifying Chemicals (CLICs) leverages spectral similarity—expressed through direct or reverse match factors between the reference spectrum in the template and the measured spectrum of a detected peak—to validate or reject matches [36, 37]. This dual criterion, combining chromatographic and spectral agreement, greatly improves the robustness of feature recognition. Fusion-based template matching thus minimizes false positives—filtering out spurious FID peaks lacking consistent MS confirmation—and reduces false negatives, as imperfect spectral matches can still be supported by the complementary FID signal.

**Fig. 5:**
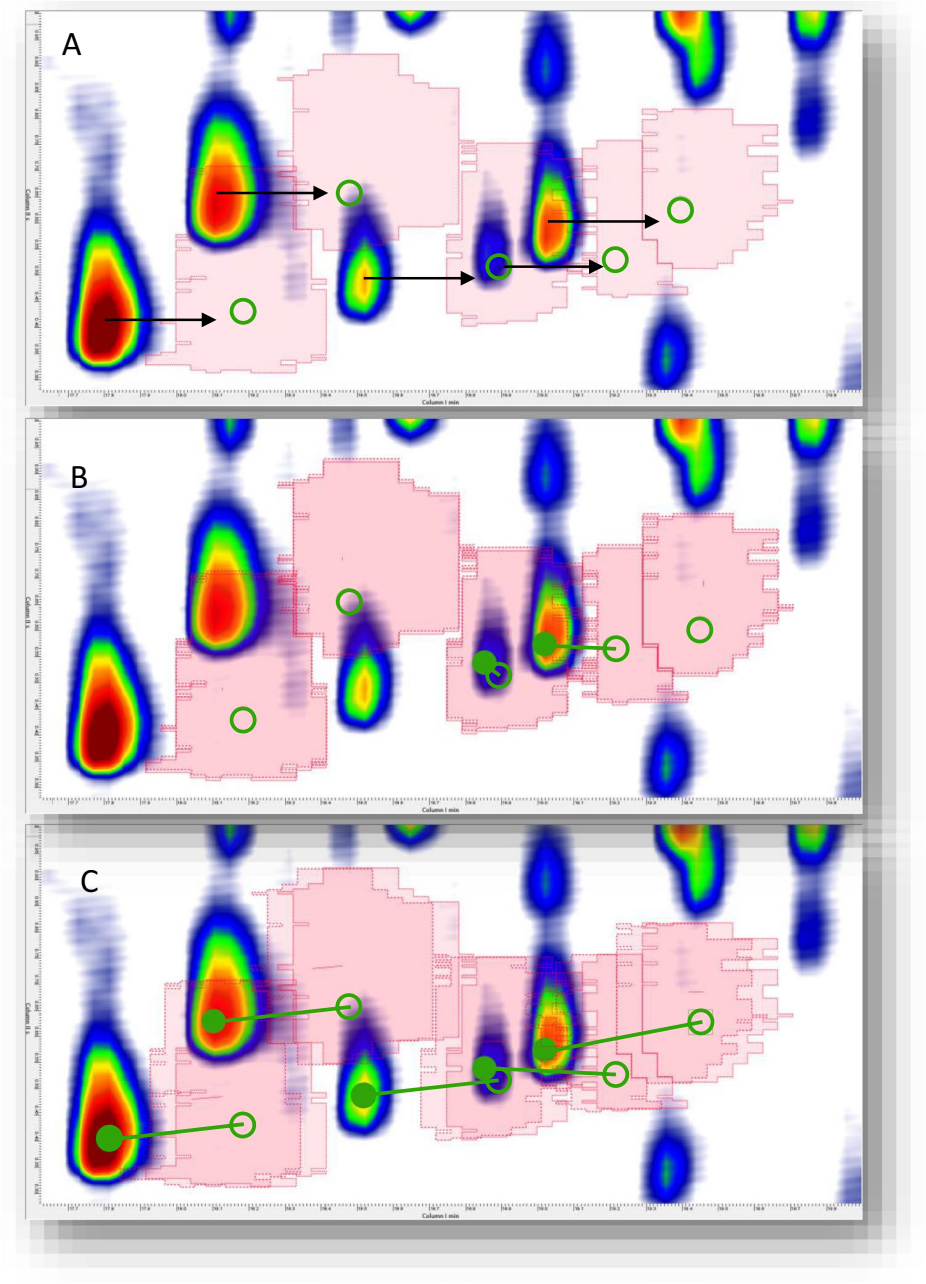
Enlarged section of the hazelnut volatiles chromatogram illustrating the retention time shift between the feature template and the actual signal traces (**A**). In **B**, the template is overlaid on the FID trace together with some mismatched peaks. **C** The fused chromatogram, where the MS spectral information embedded within the peak objects guides the alignment through spectral constraints, enabling accurate template translation and reliable matching

An example of this process is presented in Fig. 5, which illustrates a shift between the template and the chromatogram (Fig. 5A). When relying solely on the FID trace, the matching is based exclusively on temporal coordinates, which can lead to mismatches or even false positives (Fig. 5B). In contrast, in the case of fused chromatograms, the MS trace embedded within the peak object guides the matching through the applied spectral constraints (Fig. 5C). As a result, the template is properly transformed, and the match becomes more reliable.

**Fig. 6:**
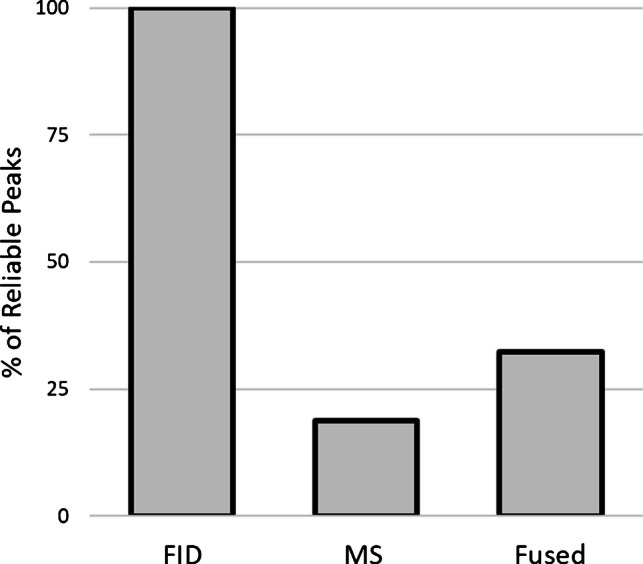
Histogram comparing the percentage of reliable peaks in the FID (reference), MS, and fused channels, processed by UT fingerprinting. The MS channel accounts for 20.8% of the FID reference (20 reliable peaks), while the fused trace reaches 32.3% (31 reliable peaks)

To substantiate these considerations, a set of hazelnut samples collected over a 12-month period was analyzed at three time points: immediately after harvest, after six months, and after twelve months of storage, for a total of 30 chromatographic analyses. All chromatographic data from these analyses were jointly processed to extract three parallel data streams: the FID chromatograms, the MS chromatograms, and the fused chromatograms. Chromatographic fingerprinting was then applied to each stream through the generation of a combined untargeted and targeted (UT) template, following the approach proposed by Stilo et al*.* [25]. After refining the preprocessing parameters according to that methodology (including optimization of signal-to-noise and volume-to-noise detection thresholds, spectral similarity constraints, reference spectrum selection, distance thresholds in both chromatographic dimensions, and polynomial transformation parameters for pattern alignment [38]), the fingerprinting effectiveness among the two detector channels and the fused data was on the reliable peaks. Reliable peaks are those peak features that are consistently detected and matched across the chromatograms (*n* = 30) according to specific matching constraints. In this work, the “most relaxed” setting for the UT fingerprinting was selected, which considers a peak as reliable when it is matched in at least half of the chromatograms/images [39]. The percentage of reliable peaks identified for each detector stream, taking the FID as a reference, is shown in Fig. 6.

The average number of peaks detected with a S/N greater than 100 ranged from 200 to 500 across the three channels. The FID trace yielded 96 reliable peaks (i.e., defined as 100%) out of approximately 500 detectable. Upon applying the mass constraint (DMF threshold > 700), the number of reliable peaks in the MS trace decreased to 20 (20.8%) out of an average of 200 detectable peaks per chromatogram. This reduction likely reflects the effective removal of false positives, although the influence of the split ratio (FID:MS = 70:30) on the absolute MS sensitivity cannot be excluded.

The fused trace, which integrates metadata from both channels, effectively compensates for the non-specific matching inherent to FID detection and for the sensitivity losses associated with MS. As a result, the number of reliable peaks increased to 31 (32.3%), while minimizing false-positive matches attributable to the limited specificity of the FID. These results demonstrate that data fusion constitutes an effective strategy for generating a reliable and consistent set of peaks, thereby enhancing the robustness of chromatographic fingerprinting and chromatogram re-alignment over extended analytical time frames.

## Conclusions

The fusion of chromatographic signals from FID and MS detectors in GC × GC represents a significant step toward the integration of complementary analytical dimensions within a single, coherent data structure. Beyond providing simultaneous access to quantitative and qualitative information, chromatogram-level fusion enhances the possibility to explore all informational dimensions concurrently, strengthening feature registration and facilitating the extraction of chemically meaningful patterns. This multidimensional representation enables a more reliable comparison of chromatographic images in the framework of Computer Vision and Augmented Visualization [40], allowing for deeper and more interpretable analyses of complex volatilomic datasets. This approach is consistent with current trends in analytical chemistry that emphasize the integration of complementary detection modes, in which quantitative robustness and qualitative selectivity are addressed jointly to enhance overall analytical reliability [7, 41].

The results of this study demonstrate that, once proper synchronization between detectors is achieved, the fused chromatogram effectively overcomes the inherent limitations of dual-detector workflows. The integration of FID and MS signals within a single data stream eliminates redundant processing, reduces interpretation errors, and substantially improves feature matching accuracy by lowering both false-positive and false-negative rates in template-based analyses. From a quantitative standpoint, the fused signal preserves the extensive linear dynamic range and reproducibility of FID while incorporating the selectivity and discriminative power of MS. This dual advantage ensures more comprehensive, scalable, and automation-ready quantification across wide concentration ranges. Overall, FID/MS chromatogram fusion establishes a robust analytical framework for high-throughput volatilomics, offering enhanced reliability, scalability, and interpretability. In particular, within the fragrances and flavours domain, this strategy provides a valuable foundation for the advancement of AI-smelling concepts [42], supporting automated odor-based classification and compliance with regulatory requirements such as allergen monitoring in consumer products.

## Supplementary Information

Below is the link to the electronic supplementary material.

Supplementary File 1 (DOCX 35.2 KB)

## Data Availability

The raw analytical datasets used for the development of the proposed data-fusion procedure are available from the corresponding author upon request.
