# Supplementary material for: Chromatogram-level fusion of FID and MS signals in GC × GC for quantitative volatilomics: workflow design and impact on pattern recognition
Source: Anal Bioanal Chem. 2026 Feb 20;418(7):2027–44. doi: 10.1007/s00216-026-06324-5 (PMC12999788; doi:10.1007/s00216-026-06324-5)
Supplement: Supplementary file 1 — (DOCX 35.2 KB) [file 216_2026_6324_MOESM1_ESM.docx]

**Supplementary Electronic Material**

**Chromatogram-Level Fusion of FID and MS Signals in GC×GC for Quantitative Volatilomics: Workflow Design and Impact on Pattern Recognition**

Andrea Caratti^1^*, Angelica Fina^1^, Fulvia Trapani^1^, Simone Squara^1^, Erica Liberto^1^, Qingping Tao^2^, Daniel Geschwender^2^, Chase Heble^2^, Stephen E Reichenbach^2,3^, Carlo Bicchi^1^, Chiara Cordero^1^*

**Supplementary Table 1** **– ST1:** Standard reference compounds used for calibration, including CAS numbers, GC–FID purity (reported as %), Target Ion (TI), Qualifier Ion 1 (Q1), and Qualifier Ion 2 (Q2).

| Compound Name | CAS-number | % FID purity | Ti | Q1 | Q2 | |
| --- | --- | --- | --- | --- | --- | --- |
| 6-Methylcoumarine | 92-48-8 | 100 | 160 | 131 | 132 | |
| Acetylcedrene | 32388-55-9 | 76.73 | 161 | 231 | 119 | |
| α - Santalol | 115-71-9 and 77-42-9 resp | 56.78 | 93 | 202 | 107 | |
| α - Terpineol | 98-55-5- | 59.38 | 136 | 121 | 93 | |
| α -Damascone (*Z*) | 43052-87-5 / 23726-94-5 | 94.04 | 192 | 123 | 69 | |
| α -Isomethylionone | 127-51-5 | 93.42 | 135 | 107 | 150 | |
| α -Pinene | 80-56-8 | 100 | 93 | 91 | 136 | |
| Amyl Cinnamal (*E*) | 122-40-7 | 100 | 202 | 129 | 115 | |
| Amyl Cinnamyl Alcohol (*E*) | 101-85-9 | 66.88 | 133 | 91 | 204 | |
| Amyl Salicylate | 2050-08-0 | 100 | 120 | 138 | 208 | |
| Anethole | 4180-23-8 | 100 | 148 | 147 | 117 | |
| Anise Alcohol | 105-13-5 | 100 | 138 | 137 | 109 | |
| Benzaldehyde | 100-52-7 | 100 | 105 | 106 | 77 | |
| Benzyl Alcohol | 100-51-6 | 100 | 79 | 108 | 107 | |
| Benzyl Benzoate | 120-51-4 | 100 | 105 | 212 | 91 | |
| Benzyl Cinnamate | 103-41-3 | 100 | 131 | 192 | 91 | |
| Benzyl Salicylate | 118-58-1 | 100 | 91 | 228 | 65 | |
| β - Santalol | 115-71-9 and 77-42-9 resp | 27.05 | 94 | 122 | 79 | |
| β -Caryophillene | 87-44-5 | 99.26 | 91 | 133 | 204 | |
| β -Damascone (*Z*) | 23726-92-3 | 95.4 | 177 | 192 | 123 | |
| β -Pinene | 127-91-3 | 100 | 93 | 79 | 69 | |
| Butylphenyl Methylproprional (Lilial) | 80-54-6 | 100 | 89 | 204 | 147 | |
| Camphor | 76-22-2 / 464-49-3 | 98.6 | 95 | 152 | 108 | |
| Carvone | 99-49-0 / 6485-40-1 / 2244-16-8 | 100 | 82 | 150 | 93 | |
| Cinnamal | 104-55-2 | 100 | 131 | 132 | 103 | |
| Cinnamyc Alcohol | 104-54-1 | 100 | 92 | 134 | 115 | |
| Citronellol | 106-22-9 / 1117-61-5 / 7540-51-4 | 100 | 69 | 41 | 156 | |
| Coumarin | 91-64-5 | 100 | 146 | 118 | 89 | |
| Damascenone | 23696-85-7 | 97.11 | 69 | 121 | 190 | |
| δ-Damascone | 57378-68-4 | 92.41 | 192 | 123 | 69 | |
| Dimethylbenzyl Carbinyl Acetate (DMBCA) | 151-05-3 | 100 | 132 | 117 | 91 | |
| Ebanol 1 | 67801-20-1 | 47.9 | 149 | 69 | 55 | |
| Ebanol 2 | 67801-20-1 | 45.9 | 149 | 83 | 93 | |
| Eugenol | 97-53-0 | 100 | 164 | 149 | 131 | |
| Eugenyl Acetate | 93-28-7 | 100 | 164 | 206 | 149 | |
| Farnesol 1 | 4602-84-0 | 48.42 | 69 | 191 | 81 | |
| Farnesol 2 | 4602-84-0 | 49.02 | 69 | 81 | 93 | |
| Geranial | 5392-40-5 | 49.21 | 69 | 152 | 84 | |
| Geraniol | 106-24-1 | 100 | 69 | 136 | 123 | |
| Geranyl Acetate | 105-87-3 / | 100 | 69 | 136 | 121 | |
| Hexadecanolactone | 109-29-5 | 100 | 55 | 236 | 41 | |
| Hexamethylindanopyran  (Galaxolide) (Musk G) | 76801-05-5 | 75.64 | 213 | 228 | 128 | |
| Hexil Cinnamal (*E*) | 101-86-0 | 93.49 | 216 | 129 | 117 | |
| Hydroxycitronellal | 107-75-5 | 100 | 59 | 43 | 71 | |
| Isoeugenol (*E*) | 97-54-1 | 94.99 | 164 | 149 | 103 | |
| Isoeugenyl Acetate | 93-29-8 | 100 | 164 | 149 | 206 | |
| Limonene | 138-86-3 | 100 | 68 | 67 | 136 | |
| Linalool | 78-70-6 | 100 | 71 | 93 | 121 |  |
| Linalyl Acetate | 115-95-7 | 100 | 93 | 136 | 121 |  |
| Lyral 1 | 31906-04-4 / 51414-25-6 | 26 | 136 | 93 | 59 |  |
| Lyral 2 | 31906-04-4 / 51414-25-6 | 74 | 136 | 93 | 59 |  |
| Menthol | 1490-04-6 / 89-78-1 / 2216-51-5 | 100 | 81 | 71 | 95 |  |
| Methyl 2-octinoate | 111-12-6 | 100 | 95 | 123 | 79 |  |
| Methyl Salycilate | 119-36-8 | 100 | 120 | 152 | 92 |  |
| Neral | 5392-40-5 | 43.34 | 69 | 41 | 134 |  |
| Propylidene Phtalide | 17369-59-4 | 92.42 | 159 | 174 | 104 |  |
| Salicylaldehyde | 90-02-8 | 100 | 122 | 121 | 65 |  |
| Sclareol | 515-03-7 | 100 | 69 | 191 | 177 |  |
| Terpinolene | 586-62-9 | 27.8 | 93 | 121 | 136 |  |
| Tetramethyl Acetyloctahydronaphtalenes (ISO & SUPER) | 54464-57-2 | 64 | 191 | 119 | 43 |  |
| Trimethyl-Benzenepropanol (Majantol) | 103694-68-4 | 83.41 | 106 | 178 | 91 |  |
| Vanillin | 121-33-5 | 100 | 151 | 81 | 152 |  |

**Supplementary Table 2 *–* ST2**. Calibration curves for the different analytes across the three instrumental set-ups, for both FID and MS detectors, including the corresponding coefficients of determination (*R²*), obtained within the concentration range of 1-100 mg/L.

|  | GC×GC flow FID/QMS | | | | GC×GC thermal FID/TOFMS Tandem ionization | | | | | | GC×2GC thermal FID/QMS | | | |
| --- | --- | --- | --- | --- | --- | --- | --- | --- | --- | --- | --- | --- | --- | --- |
|  | **FID** | | **MS** | | **FID** | | **MS 70eV** | | **MS 12eV** | | **FID** | | **MS** | |
|  | **1-100 mg/L** |  | **1-100 mg/L** |  | **1-100 mg/L** |  | **1-100 mg/L** |  | **1-100 mg/L** |  | **1-100 mg/L** |  | **1-100 mg/L** |  |
|  | **Calibration curve** | **R^2^** | **Calibration curve** | **R^2^** | **Calibration curve** | **R^2^** | **Calibration curve** | **R^2^** | **Calibration curve** | **R^2^** | **Calibration curve** | **R^2^** | **Calibration curve** | **R^2^** |
| Benzaldehyde | y = 0.10x + 3.3E-4 | 0.998 | y = 0.42x - 1.7E-2 | 0.994 | y = 0.41x - 2.5 | 1.000 | y = 0.04x + 9.9E-1 | 0.988 | y = 0.08x + 7.6E-1 | 1.000 | y = 0.07x + 3.2E-3 | 0.999 | y = 0.58x - 3.7E-3 | 0.995 |
| Limonene | y = 0.18x + 5.4E-3 | 0.996 | y = 0.52x - 3.8E-2 | 0.990 | y = 0.56x - 2.9 | 1.000 | y = 0.09x + 4.0E-1 | 0.999 | y = 0.13x - 1.6E-1 | 0.999 | y = 0.05x + 3.9E-4 | 1.000 | y = 0.59x + 1.1E−02 | 0.996 |
| Citronellol | y = 0.12x - 5.0E-3 | 0.998 | y = 0.18x - 1.8E-3 | 0.993 | y = 0.42x - 2.5 | 1.000 | y = 0.06x + 5.2E-1 | 0.998 | y = 0.08x - 6.0E-2 | 0.999 | y = 0.01x - 2.0E-3 | 0.999 | y = 0.44x - 8.7E-3 | 0.996 |
| Cinnamyc Alcohol | y = 0.08x - 8.5E-3 | 0.997 | y = 0.22x - 6.0E-2 | 0.998 | y = 0.41x - 2.5 | 0.999 | y = 0.05x + 3.4E-1 | 0.998 | y = 0.11x - 2.9E-1 | 1.000 | y = 0.02x - 9.8E-3 | 0.995 | y = 0.400x - 1.4E-2 | 0.997 |
| Eugenol | y = 0.11x - 4.7E-3 | 0.998 | y = 0.18x - 1.8E-2 | 0.998 | y = 0.38x - 1.9 | 1.000 | y = 0.07x - 4.7E-1 | 0.998 | y = 0.15x - 5.8E-2 | 0.997 | y = 0.04x - 1.1E-2 | 0.998 | y = 0.51x - 8.4E-3 | 0.996 |
| β-Damascone (*Z*) | y = 0.15x - 9.6E-4 | 0.998 | y = 0.32x - 4.9E-2 | 0.991 | y = 0.48x - 2.5 | 0.999 | y = 0.06x + 4.0E-1 | 0.998 | y = 0.12x + 6.2E-1 | 0.999 | y = 0.11x - 1.4E-2 | 0.999 | y = 0.42x - 6.9E-4 | 0.995 |
| Camphor | y = 0.14x - 3.5E-4 | 0.998 | y = 0.41x - 4.0E-3 | 0.997 | y = 0.44x - 2.6 | 1.000 | y = 0.05x + 6.9E-1 | 0.996 | y = 0.08x + 1.2E-1 | 0.995 | y = 0.03x + 1.2E-3 | 0.999 | y = 0.61x + 1.8E-2 | 0.993 |
| Isoeugenol (*E*) | y = 0.08x - 3.4E-3 | 0.998 | y = 0.17x - 1.94E-2 | 0.998 | y = 0.35x - 1.8 | 1.000 | y = 0.07x + 5.9E-3 | 1.000 | y = 0.17x + 7.0E-2 | 0.998 | y = 0.06x - 1.8E-2 | 0.996 | y = 0.51x - 1.2E-2 | 0.996 |
| Coumarin | y = 0.07x - 4.3E-3 | 0.997 | y = 0.34x - 6.0E-2 | 0.995 | y = 0.34x - 1.7 | 1.000 | y = 0.08x + 4.1E-1 | 0.996 | y = 0.12x + 1.1E-3 | 0.998 | y = 0.10x - 1.0E-2 | 1.000 | y = 0.46x - 1.0E-2 | 0.997 |
| α-Santalol | y = 0.15x - 3.2E-3 | 0.996 | y = 0.25x - 3.1E-2 | 0.994 | y = 0.52x - 2.3 | 0.998 | y = 0.09x + 3.3E-1 | 0.999 | y = 0.13x - 2.7E-1 | 1.000 | y = 0.02x - 5.8E-3 | 0.997 | y = 0.34x - 8.2E-3 | 0.999 |
| Benzyl benzoate | y = 0.12x - 1.6E-3 | 0.997 | y = 0.28x - 1.3E-2 | 0.997 | y = 0.43x - 2.3 | 0.999 | y = 0.07x + 4.2E-1 | 0.999 | y = 0.11x - 7.7E-2 | 1.000 | y = 0.10x - 1.6E-2 | 0.999 | y = 1.01x - 1.1E-2 | 0.995 |
| Benzyl cinnamate | y = 0.11x - 2.1E-4 | 0.997 | y = 0.12x + 1.3E-2 | 0.998 | y = 0.35x - 1.9 | 1.000 | y = 0.07x + 4.51E-1 | 0.998 | y = 0.10x + 4.9E-1 | 0.994 | y = 0.07x - 2.2E-2 | 0.995 | y = 0.42x - 3.3E-2 | 0.996 |
